# Levels of Protein CoAlation Regulate Redox Signaling Events of Human Sperm Capacitation

**DOI:** 10.3390/antiox15050600

**Published:** 2026-05-09

**Authors:** Chika Onochie, Valeriy Filonenko, Ivan Gout, Cristian O’Flaherty

**Affiliations:** 1 Department of Pharmacology and Therapeutics, McGill University, Montreal, QC H3G 1Y6, Canada; 2 Department of Surgery, Urology Division, McGill University, Montreal, QC H4A 3J1, Canada; 3 The Research Institute, McGill University Health Centre, Montreal, QC H4A 3J1, Canada; 4 Department of Cell Signaling, Institute of Molecular Biology and Genetics, 03680 Kyiv, Ukraine; 5 Department of Structural and Molecular Biology, University College London, London WC1E 7JE, UK; 6 Anatomy and Cell Biology, McGill University, Montreal, QC H3A 0C7, Canada

**Keywords:** CoAlation, coenzyme A, redox signaling, protein thiol modification, spermatozoa, capacitation, decapacitation factors, male infertility, PKA substrates phosphorylation, tyrosine phosphorylation

## Abstract

Infertility is a global health problem, with male factors contributing to nearly half of all cases. Up to 30% of male infertility is classified as idiopathic, in part because routine semen analysis does not assess sperm fertilizing competence. Capacitation is a complex process that endows spermatozoa with the competence to fertilize the oocyte, and it depends on oxidant-driven phosphorylation events. These events include increased PKA substrate and tyrosine phosphorylation, which promote hyperactivated motility and the acrosome reaction. These pathways are normally restrained by decapacitation factors that must be relieved in the female reproductive tract before capacitation can proceed. Protein CoAlation is an antioxidant modification of protein thiols through a disulfide bond with coenzyme A (CoASH). We previously detected protein CoAlation in human spermatozoa and observed that its levels decline during capacitation, but its function was unknown. We hypothesized that protein CoAlation functions as a decapacitation mechanism that prevents redox signalling, enabling oxidative activation of phosphorylation events during capacitation. Using spermatozoa from healthy human donors, we leveraged subcellular fractionation, immunocytochemistry, computer-assisted sperm analysis (CASA), and immunoblotting to determine the sperm protein CoAlation profile, assess CoASH biosynthetic enzymes, and test how pharmacological modulation of CoAlation levels influences capacitation. CoAlated proteins were distributed across intracellular sperm compartments, and spermatozoa possess the CoASH biosynthetic enzymes PANK2 and CoASY, indicating an intrinsic capacity for CoAlation. Inhibition of CoASH biosynthesis reduced CoAlation and enhanced PKA substrate phosphorylation, tyrosine phosphorylation, hyperactivated motility, and the progesterone-induced acrosome reaction under capacitating conditions. Pantothenic acid supplementation increased CoAlation and suppressed these processes without impairing viability or baseline motility. These findings indicate that high levels of protein CoAlation in several protein bands are a pre-existing feature of the non-capacitated state that restrains the redox-regulated events of capacitation and that its decline is required to permit sperm capacitation. CoAlation levels may emerge as a biomarker of sperm capacitation and fertilizing competence.

## 1. Introduction

Infertility, defined as the inability to achieve pregnancy after 12 months of regular, unprotected intercourse, affects roughly one in six couples worldwide, with male factors contributing to about half of all cases [1]. Male infertility is a complex condition with multiple causes; however, an oxidative stress component is implicated in most cases [2]. In addition, up to 30 percent of male infertility cases are classified as idiopathic, a condition where semen parameters appear normal despite an underlying dysfunction [3,4]. Routine semen analysis is limited because it does not detect defects in spermatozoa’s fertilizing potential or the physiological changes they undergo in the female reproductive tract [5]. To address male infertility, there is a need to develop functional biomarkers that accurately reflect sperm fertilizing ability [6].

Capacitation is the process through which the spermatozoon attains fertilizing ability [7]. It involves a series of morphological and biochemical changes that prepare spermatozoa for fertilization. Increased production of reactive oxygen and nitrogen species (RONS) in spermatozoa promotes redox signalling necessary for capacitation [8]. Membrane remodelling through cholesterol efflux increases sperm membrane fluidity and improves receptor accessibility [9]. Fluxes in ions such as sodium and potassium, together with changes in intracellular pH, establish the membrane potential required for signalling pathways [10]. Extensive protein phosphorylation regulates the molecular switches that drive capacitation [11]. Together, these changes enable hyperactivated motility, a unique whiplash-like movement required for spermatozoa to detach from the oviductal epithelium and navigate through the oviductal fluid to reach the oocyte [12]. Then, the acrosome reaction is needed for zona pellucida (a glycoprotein matrix surrounding the oocyte) penetration and oocyte fertilization [13].

In human spermatozoa, the events that drive capacitation are tightly regulated by redox signalling. Controlled generation of RONS such as superoxide (O_2_^●−^), hydrogen peroxide (H_2_O_2_) and nitric oxide (NO), is the earliest event during sperm capacitation. These oxidants act as physiological signals that promote membrane remodelling, stimulate bicarbonate-dependent activation of soluble adenylyl cyclase, and regulate the activity of kinases and phosphatases that control the extensive protein phosphorylation required for capacitation [14]. An early increase in PKA substrates phosphorylation (pPKAS) within 30 min primes a later rise in tyrosine phosphorylation (pTYR) within 2 h of the initiation of capacitation [15]. These phosphorylation events occur within a broader signalling network that includes protein kinase C (PKC), extracellular signal-regulated kinase (ERK), and the phosphoinositide 3-kinase–protein kinase B (PI3K–AKT) pathway [16]. Through these pathways, redox signalling ensures that spermatozoa acquire hyperactivated motility and the ability to undergo the acrosome reaction required for fertilization.

Proper regulation of the onset of capacitation is crucial for successful fertilization. Premature hyperactivated motility or an early acrosome reaction can exhaust fertilizing potential and compromise viability before the sperm encounters the oocyte and are associated with male infertility [17]. This regulation is achieved through decapacitation factors. Decapacitation factors are classically present in epididymal fluid or seminal plasma and associate with the sperm membrane, maintaining a non-capacitated state by suppressing the signalling pathways required for capacitation [18,19]. Most known decapacitation factors are extracellular substances derived from the seminal plasma that bind to the sperm plasma membrane. These include semenogelin I/II, the major proteins of the human semen coagulum, which inhibit capacitation by preventing superoxide generation [20]; glycodelin-S, a highly abundant glycoprotein in human seminal plasma that coats the sperm membrane to reduce albumin-induced cholesterol efflux [21]; and substances transferred to spermatozoa via epididymosomes and prostasomes (epididymis- and prostate-derived extracellular vesicles, respectively) [22], including CRISP1 [23] and cholesterol [24], which stabilize the sperm membrane and prevent premature capacitation signalling. Despite numerous extrinsic decapacitation factors, the intrinsic inhibitory mechanisms produced and regulated within the spermatozoon remain poorly defined. Identifying such intrinsic regulators is essential for understanding how spermatozoa control the onset of capacitation and how dysregulation of this control might contribute to male infertility.

The RONS generated during capacitation promote capacitation through reversible thiol post-translational modifications (PTMs), including S-nitrosylation, sulfenylation, and disulfide bond formation that result in redox signaling [25]. However, when RONS accumulate beyond physiological levels, these oxidation products become further oxidized to higher-order species such as sulfinic and sulfonic acids, which are not readily reversible and result in protein hyperoxidation. To prevent this, cells employ low-molecular-weight (LMW) thiol molecules that can reversibly conjugate to susceptible protein cysteines via disulfide bonds, shielding them from further oxidation [26]. The most studied of these is glutathionylation, in which glutathione (GSH) forms a disulfide with protein cysteines [27]. However, human spermatozoa possess a markedly reduced cytoplasmic volume and correspondingly low intracellular GSH levels, which limit their capacity to rely on glutathionylation as a primary protective mechanism [2,28].

Coenzyme A (CoASH) is a LMW thiol molecule abundant across all life forms, whose thiol group forms high-energy thioester bonds with acyl groups, generating intermediates that support metabolism. Beyond its classical metabolic role, CoASH can reversibly conjugate to protein cysteine residues via a disulfide bond under oxidative conditions, a PTM termed protein CoAlation [29]. This modification protects protein thiols from hyperoxidation and modulates protein function. We previously detected protein CoAlation in human spermatozoa and observed a prominent CoAlated band at ~45 kDa that was less abundant under capacitating conditions [30]. However, the impact of this change in CoAlation levels on capacitation, the subcellular distribution of CoAlation in spermatozoa, and the capacity of mature spermatozoa to support CoAlation autonomously remained unknown.

In this study, we hypothesized that protein CoAlation acts as an intrinsic redox-based decapacitation mechanism in human spermatozoa and that its decline is required to permit redox-regulated phosphorylation events and acquisition of fertilizing competence. Thus, we characterized the localization of CoAlation in human spermatozoa, determined whether spermatozoa express key CoASH biosynthetic enzymes, examined how decreased CoAlation affects redox-regulated phosphorylation cascades and the functional outcomes of capacitation.

## 2. Materials and Methods

### 2.1. Materials

Rabbit polyclonal anti-phospho-PKA substrates antibody (Cat. #9621L; Cell Signalling Technology, Beverly, MA, USA) and mouse monoclonal anti-CoA antibody (developed by Dr. I. Gout, University College London, London, UK, and Dr. A. Filonenko, Institute of Molecular Biology and Genetics, Kyiv, Ukraine) were used [31]. Percoll (Cat. #GE17-0891-01), 0.2 µm nitrocellulose membranes (Cat. # GE10600004), rabbit polyclonal anti-PANK2 (HPA008440), rabbit polyclonal anti-COASY (HPA022875), anti-phosphotyrosine (clone 4G10), N-ethylmaleimide (NEM; Cat. #04259-5G), pantothenic acid (D-pantothenic acid hemicalcium salt, Cat. #21210), and Pisum sativum agglutinin conjugated with FITC (PSA-FITC; L0770) were purchased from Sigma-Aldrich (Oakville, ON, Canada). Goat anti-rabbit and goat anti-mouse secondary antibodies conjugated with Alexa Fluor 555 (#A32732 and #A32727), enhanced chemiluminescence (ECL) kit (Cat. #32106), ProLong Gold Antifade reagent (#P36934), and protease inhibitor cocktail (Cat. #78410) were obtained from Thermo Fisher Scientific (Saint-Laurent, QC, Canada). Peroxidase-conjugated goat anti-mouse (Cat. #167089) and goat anti-rabbit (Cat. #171046) secondary antibodies were purchased from Jackson ImmunoResearch (Bar Harbor, ME, USA). PANK2 inhibitor (PANK2i; Cat. #31002) was obtained from Cayman Chemical (Ann Arbor, MI, USA), and HOPan (Cat. #PANK-139729) from MedChemExpress (Monmouth Junction, NJ, USA). All other chemicals were of at least reagent-grade quality.

### 2.2. Participants and Sperm Sample Preparation

This study was approved by the Ethics Board of the Research Institute at the McGill University Health Center (RI-MUHC). All participants were between 18 and 30 years old, non-smokers, and provided informed written consent. Each experiment was carried out with samples from at least 4 different donors. Semen samples from the donors were liquefied by incubation at 37 °C for 30 min, then centrifuged at 23 °C over a 4-layer Percoll gradient (20%, 40%, 60%, and 95%). The highly motile sperm fraction (progressive motility ≥70%), obtained from the 95% layer and the 60–95% interface, was used for all the experiments. Sperm concentration was assessed with a Neubauer hemacytometer. For capacitation, spermatozoa (50 × 10^6^/mL) were incubated in Biggers–Whitten–Whittingham (BWW) medium (95 mM NaCl, 4.7 mM KCl, 1.7 mM CaCl_2_, 1.2 mM KH_2_PO_4_, 1.2 mM MgSO_4_, 20 mM HEPES, 5.6 mM glucose, 0.25 mM pyruvate, and 21.4 mM sodium lactate), supplemented with 10% fetal cord serum ultrafiltrate (FCSu) as the capacitation inducer. FCSu was obtained from the fetal cord blood bank at the Royal Victoria Hospital (Montréal, QC, Canada). FCSu was prepared using Amicon Ultra-4 filter devices (UFC8010D) with 3 kDa cut-off membranes (MilliporeSigma, Oakville, ON, Canada), as previously described [32]. FCSu induces sperm capacitation-associated changes in spermatozoa (e.g., increase of protein tyrosine phosphorylation, hyperactivation and responsiveness to acrosome reaction inducers), as other capacitation inducers system such as BSA/bicarbonate [33,34,35].

### 2.3. Subcellular Fractionation

Spermatozoa were adjusted to 250 × 10^6^ cells/mL in HEPES-buffered saline (HBS 1×; 25 mM HEPES, 14 mM fructose, 115 mM NaCl, 4 mM KCl, and 0.5 mM MgCl_2_·6H_2_O, pH 8.0), as previously described with minor modifications [36]. A protease inhibitor cocktail was added to a final concentration of 1%, and a 50 µL aliquot was set aside as the whole-sperm sample (from which 10 µL, or 2 × 10^6^ spermatozoa was loaded in the well). The remaining suspension was frozen at −80 °C for 15 min and thawed at 4 °C to disrupt membranes. Samples were centrifuged at 2000× *g* for 5 min at 4 °C, and 50 µL of the supernatant was collected as the cytosolic fraction. The pellet was resuspended in 400 µL HBS 1× containing 0.2% Triton X-100, incubated on ice for 10 min, and centrifuged at 12,000× *g* for 5 min at 4 °C. A 50 µL aliquot of this supernatant was collected as the Triton-soluble fraction. The remaining pellet was resuspended in 400 µL HBS 1×, sonicated on ice (3 × 15 s at 25% output; Vibracell, Sonics & Materials Inc., Newtown, CT, USA), and 50 µL of this suspension was collected as the Triton-insoluble fraction. To obtain head and tail fractions, the Triton-insoluble fraction was examined under a brightfield microscope (Wetzlar, Germany) (Leica DMI6000, 40×) to confirm mechanical separation, layered onto a 40%/90% Percoll gradient, and centrifuged at 2000× *g* for 5 min at 4 °C. The interphase was collected as the tail fraction, whereas the 90% layer (heads) was washed in HBS 1× and centrifuged again at 2000× *g* for 5 min to remove residual Percoll. The head pellet was resuspended in 50 µL HBS 1×. All fractions were immediately mixed with reducing (+100 mM dithiothreitol (DTT)) or non-reducing sample buffer (without DTT), heated at 90 °C for 5 min, and used for downstream SDS-PAGE.

### 2.4. SDS-PAGE and Immunoblotting

Following incubation in the corresponding treatment condition, the samples were mixed with either reducing or non-reducing sample buffer supplemented with a protease inhibitor cocktail and phosphatase inhibitors. The mixtures were heated at 95 °C for 5 min and centrifuged at 21,000× *g* for 5 min at room temperature. Supernatants containing proteins from 0.4 × 10^6^ spermatozoa were loaded into each well and then resolved on 10% polyacrylamide gels under constant current (0.025 A/gel) and electrotransferred onto nitrocellulose membranes for 45 min at 100 V. Membranes were blocked with 5% skim milk in TTBS1X for 1 h, followed by incubation with primary antibodies overnight at 4 °C. After washes in TTBS, membranes were incubated with horseradish peroxidase–conjugated secondary antibodies (goat anti-mouse or donkey anti-rabbit, 1:2500) for 1 h at room temperature. Immunoreactive bands were visualized using enhanced chemiluminescence and imaged with an Amersham Imager 600/680 (GE Healthcare, Montréal, QC, Canada). Following detection, membranes were silver-stained (2% *w/v* trisodium citrate, 0.8% *w/v* FeSO_4_, 0.2% *w/v* AgNO_3_ in deionized water). Band intensities of proteins of interest (e.g., 105 and 80 kDa for phosphotyrosine) were quantified using FIJI/ImageJ (version 2.1.0/1.53c, NIH, Bethesda, MD, USA). Densitometry values were first normalized to the corresponding silver stain loading control and then normalized to the non-capacitated control sample. Data are presented as mean ± standard error.

### 2.5. Immunocytochemistry

Immunocytochemistry was performed on methanol-permeabilized and non-permeabilized spermatozoa smeared onto Superfrost slides and air-dried. For permeabilization, slides were incubated in 100% methanol at −20 °C for 20 min, followed by rehydration in phosphate-buffered saline (PBS) containing 0.2% Triton X-100 for 20 min. Slides were blocked with 5% normal goat serum in PBS for 30 min at room temperature, then incubated overnight at 4 °C with primary antibodies against CoA (1:20), PANK2 (1:50), or COASY (1:100), diluted in blocking buffer. After washing, samples were incubated with Alexa Fluor 555-conjugated goat anti-mouse (for CoA) or goat anti-rabbit (for PANK2 and COASY) secondary antibodies (1:1000) for 1 h at room temperature. Slides were mounted with ProLong Antifade and sealed. Negative controls received secondary antibody only. Images were acquired at 63× magnification using a Leica DMI6000 fluorescence microscope.

### 2.6. Sperm Motility and Viability

Sperm motility was evaluated using computer-assisted sperm analysis (CASA). An aliquot from each treatment condition was analyzed using an IVOS II CASA system (Hamilton Thorne, Beverly, MA, USA) equipped with a 20× negative phase-contrast objective and temperature control. Sperm viability was assessed by the hypo-osmotic swelling (HOS) test. After 30 min of incubation at 37 °C in HOS solution (1.5 mM fructose, 1.5 mM sodium citrate), samples were mounted on Superfrost slides and examined under 20× magnification. At least 200 spermatozoa were evaluated per sample, and cells displaying tail curling were classified as viable [37]. Hyperactivation was characterized by curvilinear velocity (VCL) ≥ 150 µm/s, linearity (LIN) ≤ 50%, and amplitude of lateral head displacement (ALH) ≥ 7.0 µm, captured at 60 Hz, as previously described [38].

### 2.7. Assessment of Acrosome Reaction

The progesterone-induced acrosome reaction (AR) was assessed according to the method of Tamburrino et al., with minor modifications [39]. After 3.5 h of incubation under capacitating conditions in BWW medium containing 10% FCSu, non-capacitated and capacitated spermatozoa were incubated with 10 µM progesterone in BWW at 37 °C for 30 min. Samples were centrifuged at 600× *g* for 5 min, and the pellets were resuspended in 500 µL pre-warmed HOS solution and incubated for an additional 30 min at 37 °C. Cells were then centrifuged, fixed in 95% ethanol, smeared onto Superfrost slides, and air-dried. PSA-FITC (20 µL, 30 µg/mL) was applied to each smear, and slides were incubated at 37 °C for 5 min, rinsed in distilled water, air-dried, and mounted with antifade solution. Acrosomal status was evaluated by fluorescence microscopy using an inverted Leica DMI6000 microscope at 63× magnification. Spermatozoa exhibiting PSA-FITC fluorescence confined to the equatorial segment were classified as acrosome-reacted, whereas those with fluorescence over the entire acrosomal cap were considered acrosome-intact. At least 200 spermatozoa were counted per sample; HOS-induced tail curling was used to confirm viability.

### 2.8. Statistical Analysis

All data are presented as mean ± SEM. The normality of the data and the homogeneity of variances were evaluated using the Shapiro–Wilk and Levene tests, respectively. Statistical differences were analyzed using ANOVA followed by Bonferroni post hoc tests; *p* ≤ 0.05 was considered statistically significant.

## 3. Results

### 3.1. CoAlation Modifies Multiple Protein Bands Across Distinct Compartments of Human Spermatozoa

**Figure 1:**
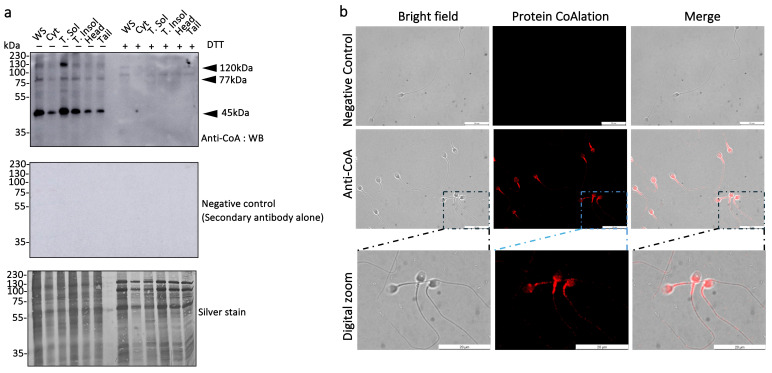
**CoAlation modifies multiple protein bands across distinct compartments of human spermatozoa.** (**a**) Sperm proteins from whole sperm (WS), cytosolic (Cyt), Triton-soluble (T-sol, membrane-associated), Triton-insoluble (T-insol, cytoskeletal/detergent-resistant), isolated head, and tail subcellular fractions were immunoblotted with anti-CoA to determine the protein CoAlated bands of human spermatozoa. CoAlated proteins were found at 45, 77, and 120 kDa. Signal is lost with DTT, confirming CoAlation is formed by a disulfide bond. A negative control blot (secondary antibody alone) showed no signal, confirming antibody specificity. Silver staining shows comparable loading between samples treated with or without DTT. (**b**) Immunocytochemistry of permeabilized spermatozoa shows robust labelling in the post-acrosomal region with signals toward the equatorial segment, strong enrichment at the midpiece, and a weaker continuous signal along the principal piece. Negative controls showed no labelling. A similar localization pattern was observed in non-permeabilized spermatozoa (Supplementary Figure S1a). Images were acquired at 63× magnification; scale bars = 20 µm.

Subcellular fractionation of human spermatozoa at 250 × 10^6^ cells/mL (corresponding to proteins from 2 × 10^6^ spermatozoa loaded per well), revealed multiple CoAlated bands by anti-CoA immunoblotting, most prominently at 45, 77, and 120 kDa (Figure 1a). CoAlation was enriched in the Triton-soluble (membrane-associated) fraction. Still, CoAlated proteins were also evident in the cytosolic and Triton-insoluble (cytoskeletal/detergent-resistant) fractions, as well as in isolated head and tail fractions. The anti-CoA signal was abolished by DTT in all fractions, confirming that the modification is mediated by disulfide bonds, while the same blot without primary antibody showed no signal, confirming the absence of non-specific binding by the secondary antibody and no spontaneous signal from the membrane. Immunocytochemistry in permeabilized spermatozoa corroborated these findings, revealing a robust CoAlation signal in the post-acrosomal region and extending towards the equatorial segment, with strong labelling in the midpiece and a weaker continuous signal along the principal piece (Figure 1b). A similar pattern was observed in non-permeabilized spermatozoa (Supplementary Figure S1a).

### 3.2. Human Spermatozoa Possess the Key Enzymes Required for CoASH Biosynthesis

**Figure 2:**
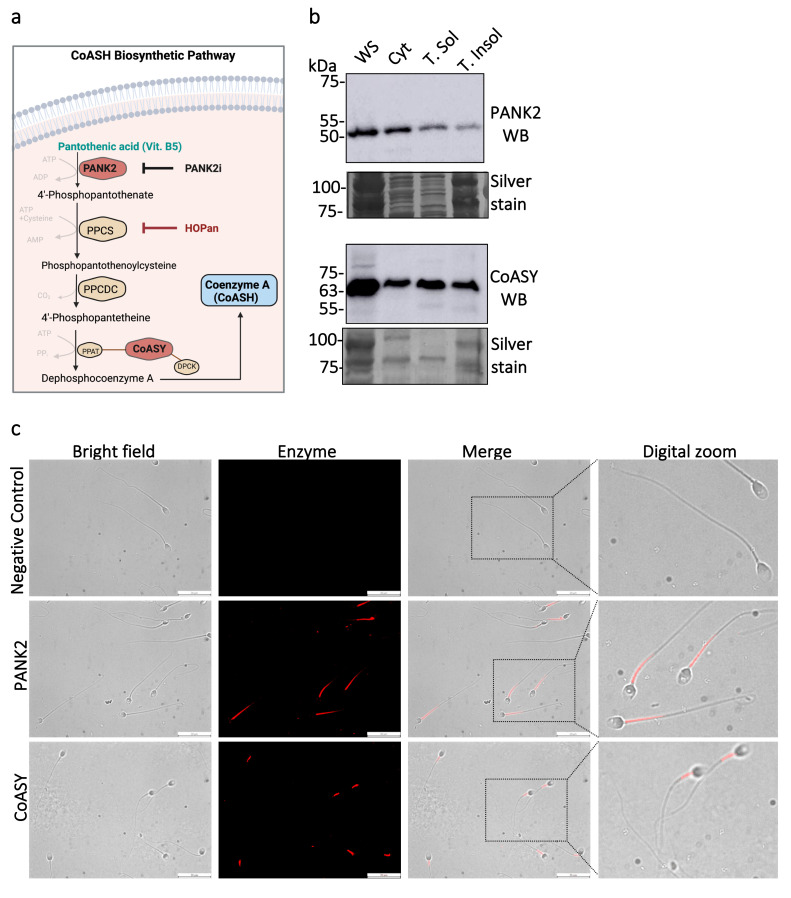
**Human spermatozoa possess the key enzymes required for CoASH biosynthesis.** (**a**) Schematic representation of the CoASH biosynthetic pathway. Pantothenic acid (vitamin B5) is converted to coenzyme A (CoASH) through a multi-step enzymatic process involving pantothenate kinase 2 (PANK2), phosphopantothenoylcysteine synthase (PPCS), phosphopantothenoylcysteine decarboxylase (PPCDC), and CoA synthase (CoASY). PANK2 catalyzes the first and rate-limiting step and is inhibited by the pharmacological agent pantothenate kinase 2 inhibitor (PANK2i). This figure was designed using BioRender (2026) https://BioRender.com/76c0a1v accessed on 30 April 2026). (**b**) Western blot analysis confirms the presence of PANK2 and CoASY. (**c**) Immunofluorescence detection of PANK2 and CoASY in spermatozoa. PANK2 localizes predominantly to the midpiece and along the flagellum, whereas CoASY is localized mainly in the midpiece. Negative control (secondary antibody only) shows no signal. Images were acquired at 63× magnification; scale bars = 20 µm.

We next asked whether mature human spermatozoa possess the enzymatic machinery required to support CoASH biosynthesis [40]. Pantothenate Kinases (PANKs) are a family of 4 isoforms (PANK1–4) that catalyze the first rate-limiting step of de novo CoASH biosynthesis. The Protein Atlas project identified all four isoforms (PANK1–4) at the transcript or protein levels [41,42,43,44]. Our study represents the first to report the identification of PANK2 at the protein level in mature human spermatozoa. Our interest in PANK2 arises from its being the most highly expressed isoform in human testis at both the transcript and protein levels, and from targeted deletion of the PANK2 gene in mice, which produces a male infertility phenotype [45]. In humans, PANK2 is the mitochondrial pantothenate kinase isoform [46]. PANK2 and CoA synthase (CoASY), which catalyze the first and final rate-limiting steps of CoASH synthesis, respectively, were readily detected by immunoblotting in whole sperm and across subcellular fractions at the expected molecular weights of 50 kDa and 63 kDa, respectively (Figure 2a,b). Immunocytochemistry localized these enzymes primarily to the flagellum (Figure 2c): PANK2 signal was highly expressed in the midpiece and extended into the principal piece, while CoASY labelling was largely confined to the midpiece. Negative controls showed no labelling. A similar pattern was observed in non-permeabilized spermatozoa (Supplementary Figure S1b).

### 3.3. Modulation of CoASH Biosynthesis Regulates Protein CoAlation Levels During Capacitation

**Figure 3:**
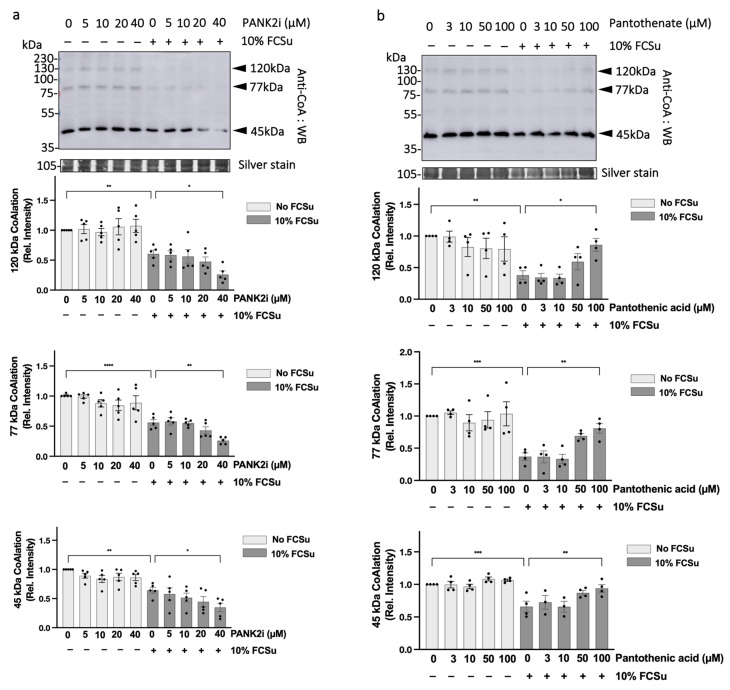
**Modulation of CoASH biosynthesis regulates protein CoAlation levels during capacitation.** Spermatozoa were incubated for 30 min at 37 °C with (capacitating conditions) or without 10% FCSu in the presence of increasing concentrations of either PANK2 inhibitor (PANK2i; 0–40 µM, panel (**a**)) or pantothenic acid (0–100 µM, panel (**b**)). Protein CoAlation was assessed by immunoblotting with anti-CoA antibody under non-reducing conditions. Relative intensity of the most prominent protein CoAlated bands (120 kDa, 77 kDa, and 45 kDa) was determined by optical protein densitometry, normalized first to the respective silver stain loading control under reducing conditions, and then to the non-capacitated control without any treatment. (**a**) PANK2 inhibition further decreased CoAlation at all three molecular weights compared to capacitating controls (10% FCSu alone). (**b**) In contrast, supplementation with pantothenic acid concentration-dependently increased CoAlation levels at all three bands, reversing the FCSu-induced decline. Data are presented as mean ± SEM; individual points represent different donors (n = 5). Statistical analysis was performed using ANOVA with Bonferroni post hoc analysis. Significant differences are indicated as * *p* < 0.05, ** *p* < 0.01, *** *p* < 0.001, **** *p* < 0.0001.

Because protein CoAlation levels decline during capacitation, we investigated whether altering intracellular CoASH biosynthesis would modulate the levels of this protein modification. We capacitated spermatozoa with 10% FCSu for 30 min, a time corresponding to the earliest time at which a significant decrease in CoAlation is observed [30]. Following incubation at 50 × 10^6^ cells/mL, spermatozoa were concentrated by centrifugation to 100 × 10^6^ cells/mL prior to lysis (corresponding to proteins from 0.8 × 10^6^ spermatozoa loaded per well), allowing us to observe multiple CoAlated bands at 120, 77, and 45 kDa compared with our previous work [30]. Under these conditions, capacitation alone reduced CoAlation of the 120, 77, and 45 kDa bands (Figure 3).

Pharmacological modulation of CoASH biosynthesis bidirectionally altered this pattern: PANK2 inhibition caused a further, concentration-dependent decrease in CoAlation at all three bands, whereas pantothenic acid supplementation produced a concentration-dependent increase, reversing the FCSu-induced decline (Figure 3a,b). The most robust and significant effects were observed at 40 µM PANK2i and 100 µM pantothenic acid, which were selected for subsequent experiments. These findings validate PANK2i and pantothenic acid as effective tools for manipulating CoAlation levels during capacitation.

### 3.4. Modulation of CoAlation Levels Does Not Impair Sperm Viability or Motility

**Figure 4:**
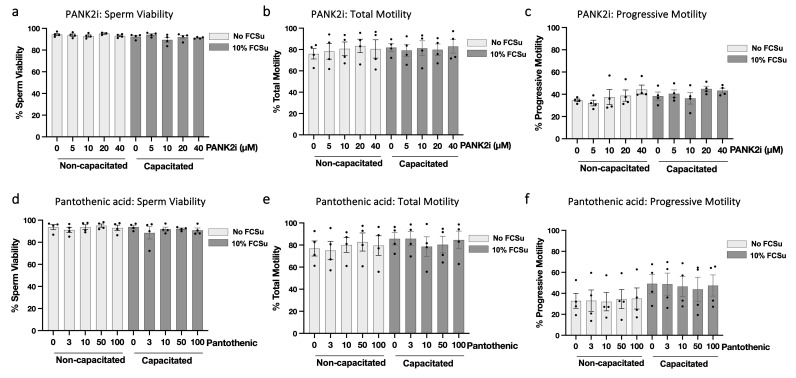
**Modulation of protein CoAlation levels does not impair sperm viability or motility.** Spermatozoa were treated with increasing concentrations of PANK2 inhibitor (PANK2i; 0–40 µM) or pantothenic acid (0–100 µM) under non-capacitating or capacitating (10% FCSu) conditions for 30 min at 37 °C. Sperm viability remained above 80% across all conditions following treatment with PANK2i (**a**) or pantothenic acid (**d**), indicating no cytotoxic effects. Total and progressive motility was unaffected by PANK2i (**b**,**c**) or pantothenic acid (**e**,**f**) under both non-capacitating and capacitating conditions. These data confirm that pharmacological modulation of CoASH biosynthesis does not compromise sperm viability or motility. Data are presented as mean ± SEM; individual data points represent different donors (n = 4). Data were analyzed using ANOVA followed by Bonferroni post hoc test. No statistically significant differences were observed between groups.

To ensure that the observed changes in CoAlation were not secondary to toxicity, we evaluated sperm viability and motility after treatment with PANK2i or pantothenic acid under non-capacitating and capacitating conditions. Across all concentrations tested, sperm viability remained above 80% and was not altered by either compound (Figure 4a,d). Similarly, total and progressive motility were unchanged in both media, indicating that neither PANK2 inhibition nor pantothenic acid supplementation affected basal motility parameters (Figure 4b,c,e,f). These data confirm that the concentrations used to modulate CoAlation are non-toxic validate PANK2i and pantothenic acid as effective tools for manipulating CoAlation levels during capacitation.

### 3.5. Low Levels of Protein CoAlation Enhance Sperm PKA Substrate Phosphorylation (pPKAS)

**Figure 5:**
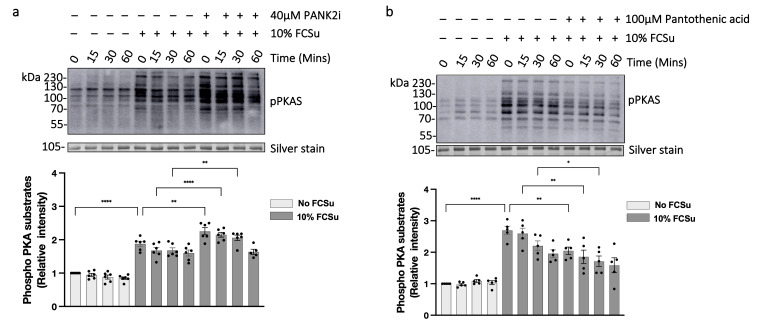
**Low levels of protein CoAlation enhance sperm PKA substrate phosphorylation (pPKAS).** (**a**) Representative immunoblot showing phospho-PKA substrates (pPKAS) levels in human spermatozoa incubated under non-capacitating and capacitating conditions (10% FCSu) with or without 40 µM PANK2 inhibitor (PANK2i) over the course of 60 min at 37 °C. PANK2i treatment, which lowers protein CoAlation levels, amplified the intensity of pPKAS during capacitation compared to sperm capacitated without any treatment. (**b**) Representative immunoblot of pPKAS in spermatozoa incubated under non-capacitating and capacitating (10% FCSu) conditions with or without 100 µM pantothenic acid over the course of 60 min. Pantothenic acid, which elevates protein CoAlation, reduced the intensity of pPKAS during capacitation compared to sperm capacitated without any treatment. Densitometric quantification of pPKAS levels was determined by assessing optical protein density, normalized first to the respective silver stain loading control and then to the non-capacitated control without any treatment, and presented as mean ± SEM; individual data points represent different donors (n = 5–6). Data were analyzed using ANOVA followed by Bonferroni post hoc test. Significant differences are indicated as * *p* < 0.05, ** *p* < 0.01, **** *p* < 0.0001.

We first examined whether decreased protein CoAlation influences early redox-regulated phosphorylation events by assessing PKA substrate phosphorylation (pPKAS). In spermatozoa incubated in BWW supplemented with 10% FCSu, pPKAS increased rapidly, peaked after 30 min, and declined thereafter. Lowering CoAlation with 40 µM PANK2i consistently amplified pPKAS intensity at all times compared with FCSu-only treated controls (Figure 5a). In contrast, elevating CoAlation with 100 µM pantothenic acid attenuated the FCSu-induced increase in pPKAS (Figure 5b). To corroborate these findings using an inhibitor targeting a different step in the CoASH biosynthetic pathway, we treated spermatozoa with HOPan, a pantothenic acid analogue that blocks PPCS [47]. At concentrations that did not impair sperm viability or motility (Supplementary Figure S2a–e), HOPan similarly increased pPKAS during capacitation (Supplementary Figure S2f). Together, these findings indicate that reduced protein CoAlation enhances PKA-dependent phosphorylation during the early phase of capacitation.

### 3.6. Low Levels of Protein CoAlation Enhance Sperm Protein Tyrosine Phosphorylation (pTYR)

**Figure 6:**
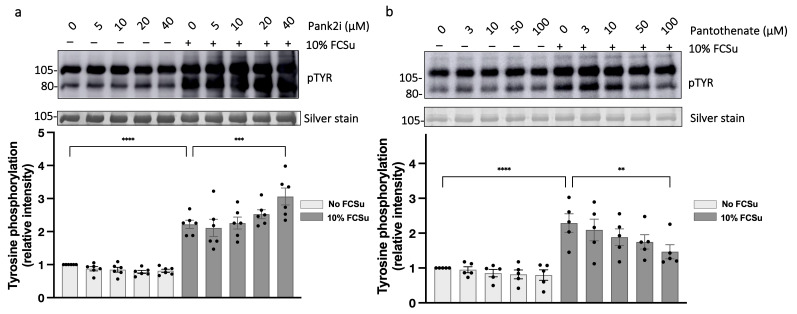
**Low levels of protein CoAlation enhance sperm protein tyrosine phosphorylation (pTYR).** (**a**) Representative immunoblot showing tyrosine-phosphorylated proteins in sperm incubated under capacitating conditions (10% FCSu, 3.5 h) with increasing concentrations of PANK2 inhibitor (PANK2i; 0–40 µM). pTYR levels increased concentration-dependently with PANK2i treatment during capacitation. (**b**) Representative immunoblot of sperm incubated with increasing concentrations of pantothenic acid (0–100 µM) under the same capacitating conditions. Elevated pantothenic acid levels suppressed the pTYR signal during capacitation. Densitometric quantification of pTYR levels was determined by assessing optical protein density of the 105 kDa and 80 kDa bands, normalized first to the respective silver stain loading control and then to the non-capacitated control without any treatment. Data are presented as mean ± SEM; individual data points represent different donors (n = 6 for panel (**a**); n = 5 for panel (**b**)). Data were analyzed using ANOVA followed by Bonferroni post hoc test. Significant differences are indicated as ** *p* < 0.01, *** *p* < 0.001, **** *p* < 0.0001.

We next assessed the impact of CoAlation on downstream redox-regulated phosphorylation by measuring protein tyrosine phosphorylation (pTYR), a late event during capacitation. After 3.5 h in 10% FCSu, capacitated spermatozoa showed a marked increase in pTYR compared with non-capacitated controls (Figure 6a). Inhibition of PANK2 further augmented pTYR in a concentration-dependent manner, indicating that lowering CoAlation potentiates capacitation-associated tyrosine phosphorylation. Conversely, pantothenic acid supplementation led to a concentration-dependent reduction in pTYR levels in FCSu-treated spermatozoa (Figure 6b). Consistent with the effects of PANK2i, HOPan increased pTYR levels during capacitation (Supplementary Figure S2g). These results demonstrate that low CoAlation levels facilitate the late tyrosine phosphorylation events that characterize capacitation.

### 3.7. Low Levels of Protein CoAlation Enhance Sperm Hyperactivated Motility

**Figure 7:**
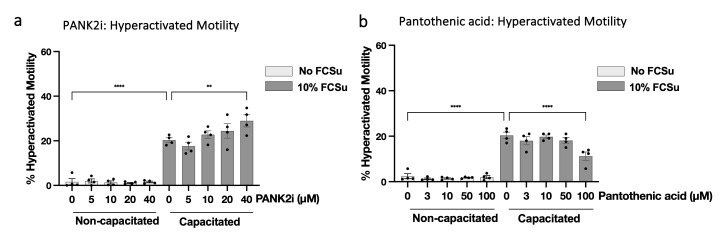
**Low levels of protein CoAlation enhance sperm hyperactivated motility.** Bar graphs showing the quantification of hyperactivated motility (% hyperactivated cells) in spermatozoa treated with increasing concentrations of PANK2 inhibitor (**a**) or pantothenic acid (**b**) under non-capacitating or capacitating conditions (10% FCSu). Capacitation significantly increased hyperactivated motility compared to non-capacitated controls. PANK2i treatment further enhanced hyperactivation in a concentration-dependent manner, while pantothenic acid supplementation suppressed the capacitation-induced hyperactivation. Data are presented as mean ± SEM; individual data points represent different donors (n = 4). Data were analyzed using ANOVA followed by Bonferroni post hoc test. Significant differences are indicated as ** *p* < 0.01, **** *p* < 0.0001.

Because capacitation is associated with the acquisition of hyperactivated motility, we asked whether changes in CoAlation influence this functional readout. CASA showed that capacitation in 10% FCSu increased the proportion of hyperactivated sperm compared with non-capacitated controls (Figure 7 and Supplementary Figure S1c). Lowering CoAlation by PANK2 inhibition further increased the percentage of hyperactivated cells beyond that induced by capacitation alone, whereas elevating CoAlation with pantothenic acid reduced hyperactivation in a concentration-dependent manner. HOPan treatment, also enhanced the percentage of hyperactivated spermatozoa (Supplementary Figure S2a,e). These findings indicate that reduced protein CoAlation allows the acquisition of hyperactivated motility during capacitation.

### 3.8. Low Levels of Protein CoAlation Enhance Progesterone-Induced Acrosome Reaction

**Figure 8:**
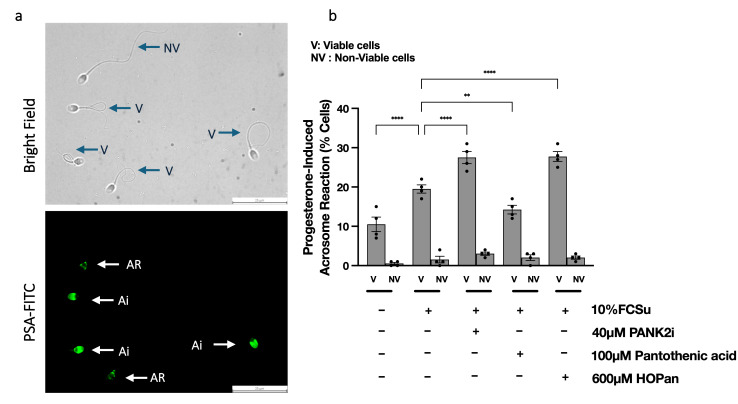
**Low levels of protein CoAlation enhance the progesterone-induced acrosome reaction.** (**a**) Representative brightfield and PSA–FITC images showing viable (V) and non-viable (NV) spermatozoa. PSA–FITC staining distinguished intact acrosomes (Ais) from reacted acrosomes (ARs). Scale bar = 25 µm. (**b**) Quantification of the acrosome reaction in viable (V) and non-viable (NV) sperm following 3.5 h incubation at 37 °C under the following conditions: non-capacitated (No FCSu), capacitated with 10% FCSu, capacitated with 10% FCSu plus 40 µM PANK2 inhibitor (PANK2i), 100 µM pantothenic acid or 600 µM HOPan. After incubation, each sample was further incubated for 30 min at 37 °C with or without 10 µM progesterone. Viable sperm capacitated in the presence of CoASH biosynthesis inhibitors (PANK2i and HOPan) exhibited a higher percentage of acrosome-reacted cells compared to non-treated capacitated controls, whereas those in the presence of pantothenic acid had lower proportion of acrosome-reacted cells. Non-viable sperm showed minimal acrosome reaction across all conditions. Data are presented as mean ± SEM; individual data points represent different donors (n = 4). Data were analyzed using ANOVA followed by Bonferroni post hoc test. Significant differences are indicated as ** *p* < 0.01, **** *p* < 0.0001.

Finally, we evaluated whether modulating CoAlation affects the progesterone-induced acrosome reaction (AR), the terminal functional outcome of capacitation and a state indicating that the spermatozoon is fully capacitated. Capacitation in 10% FCSu increased the proportion of acrosome-reacted sperm within the viable population compared with non-capacitated controls, while non-viable cells showed minimal responsiveness under all conditions (Figure 8). Treatment with 40 µM PANK2i or 600 µM HOPan, both of which are inhibitors of CoASH biosynthesis, further increased the percentage of viable sperm undergoing progesterone-induced AR, whereas 100 µM pantothenic acid, which is a precursor for CoASH biosynthesis, significantly reduced this response (Figure 8b). These results demonstrate that low protein CoAlation facilitates the functional outcome of capacitation, the progesterone-induced acrosome reaction.

## 4. Discussion

This study provides the first evidence that protein CoAlation may represent an intrinsic decapacitation mechanism in human spermatozoa, as it is present at high levels in several protein bands in non-capacitated spermatozoa and is diminished under capacitating conditions. CoAlated proteins were detected across multiple intracellular compartments, including cytosolic, membrane-associated, and detergent-resistant fractions, and in both sperm heads and tails. We also found that human spermatozoa possess the rate-limiting CoASH biosynthetic enzymes PANK2 and COASY. Lower CoAlation levels in capacitating conditions enhanced PKA substrate phosphorylation, tyrosine phosphorylation, hyperactivated motility, and the progesterone-induced acrosome reaction. In contrast, higher CoAlation suppressed these events without affecting viability or baseline motility. Altogether, these findings indicate that CoAlation in non-capacitated spermatozoa restrains redox signalling pathways, and that its decline under capacitating conditions is required to allow the acquisition of fertilizing competence by the spermatozoon.

Classical decapacitation factors, largely present in the epididymal fluid and seminal plasma, stabilize the sperm membrane and suppress the capacitation-associated signalling in spermatozoa. Despite extensive examples of extrinsic decapacitation factors, the intrinsic inhibitory mechanisms within the spermatozoon are poorly defined. NYD-SP27, an acrosomal protein whose loss during capacitation permits the acrosome reaction, provides one example of a sperm-intrinsic mechanism acting in a decapacitating manner [48]. Our data extend this concept by showing that a post-translational modification functions as an intrinsic inhibitory mechanism associated with the non-capacitated state. Here, we demonstrated that CoAlation is sperm-intrinsic: CoAlated proteins persisted in washed spermatozoa across multiple intracellular fractions, and spermatozoa independent of seminal plasma have the enzymes required for de novo CoASH biosynthesis to support CoAlation. We further provided evidence that CoAlation plays a decapacitation-like, inhibitory role, as protein CoAlation levels are higher in the non-capacitated state and are significantly decreased in capacitating conditions. Persistently high protein CoAlation levels in capacitating conditions achieved by pharmacological modulation of CoASH biosynthesis result in impaired capacitation, whereas low protein CoAlation levels significantly enhanced the outcomes of capacitation. These observations support the interpretation that protein CoAlation represents an intrinsic inhibitory mechanism that has to be removed for capacitation to proceed and are consistent with protein CoAlation serving as a novel intrinsic decapacitation mechanism in human spermatozoa.

Redox signalling is a central driver of mammalian sperm capacitation, and several key events in this process are promoted by the RONS generated during capacitation [14,15,16]. Among these redox-regulated events are phosphorylation cascades that depend on coordinated redox control of both kinases and phosphatases. Within this framework, the CoAlated protein bands at 45, 77, and 120 kDa may represent redox-sensitive proteins that are insulated from premature oxidation in non-capacitated spermatozoa. Although we did not identify the protein constituents of these bands, their approximate molecular weights are compatible with several redox-sensitive regulators of phosphorylation cascades and provide illustrative examples of how protein CoAlation might modulate these pathways. The 45 kDa band plausibly corresponds to the regulatory subunits of PKA (PKA-R) [49], where CoAlation of its redox-sensitive cysteines could limit oxidative activation of PKA and maintain low phosphorylation of PKA substrates. DeCoAlation during capacitating conditions would expose these cysteines to oxidants and facilitate PKA activation, consistent with increased PKA substrate phosphorylation when CoAlation is lowered. This band may also include Aurora A kinase (AURKA, 46 kDa). Tsuchiya et al. demonstrated that CoASH directly inhibits AURKA by competing with ADP at the ADP-binding site, both in vitro and in vivo [50]. AURKA has recently emerged as a multifunctional kinase in spermatozoa, extending beyond its canonical mitotic role. In mouse sperm, total AURKA localizes to the principal piece of the flagellum while its activated, phosphorylated form is enriched in the midpiece [51]. Proteomic analysis of AURKA-interacting partners in sperm flagella identified mitochondrial proteins such as complex V (F_1_F_0_-ATPase) subunits, as well as fibrous sheath and axonemal components [52]. These findings suggest that AURKA may regulate sperm flagellar motility. Although a direct role for AURKA in sperm capacitation has not yet been investigated, it is noteworthy that hyperactivated motility, a hallmark of capacitation, was significantly suppressed by pantothenic acid supplementation in our study. Whether the 45 kDa CoAlated protein is AURKA, and whether CoAlation and/or elevated free CoASH contribute to the observed suppression of hyperactivated motility through inhibition of this kinase, warrants further investigation.

The 77 kDa band may include protein kinase C (PKC) [53,54]. CoAlation of cysteine-rich C1 domains could protect against oxidative activation of PKC, thereby helping maintain low tyrosine phosphorylation in the non-capacitated state. DeCoAlation would permit oxidative activation of PKC, consistent with the enhanced protein tyrosine phosphorylation and acrosome reaction observed when protein CoAlation is reduced. Another candidate protein at this band may include c-Src (~70 kDa), a redox-sensitive non-receptor tyrosine kinase present in human sperm [55]. Src contains critical cysteine residues (Cys-185 and Cys-277) that, upon oxidation to sulfenic acids, promote kinase activation by destabilizing the autoinhibited conformation [56]. We speculate that CoAlation of these cysteines in non-capacitated spermatozoa maintains Src in an inactive state; subsequent deCoAlation during capacitation would render these residues susceptible to oxidative activation, thereby contributing to the capacitation-associated increase in tyrosine phosphorylation. The band migrating at 120 kDa could correspond to c-Abl (cellular Abelson tyrosine kinase 1), which promotes tyrosine phosphorylation during capacitation downstream of PKA signalling [57] and whose kinase activity is enhanced under oxidative conditions [58]. CoAlation of c-Abl in non-capacitated spermatozoa may maintain this kinase in an inactive state; subsequent deCoAlation during capacitating conditions would then render c-Abl susceptible to oxidative activation, thereby promoting tyrosine phosphorylation. These assignments need to be confirmed since each band likely contains multiple proteins. However, they support a model in which CoAlation acts in a decapacitation-like manner in spermatozoa, preventing redox-sensitive proteins from being oxidized until capacitating cues are present. Identifying the protein constituents and CoAlation sites within these bands will be essential to test this model.

The decline in protein CoAlation during capacitation, an oxidative process, may appear paradoxical since oxidative conditions would be expected to increase CoAlation as a protection against hyperoxidation [29]. However, a decrease in redox-dependent protein modifications during capacitation is not unique to CoAlation. Mostek-Majewska et al. demonstrated that protein S-glutathionylation levels decline during bull sperm capacitation [59]. Together, the decline in S-glutathionylation during bull sperm capacitation and the decline in CoAlation we observe here are consistent with an ordered sequential process during capacitation: removal of protective LMW thiol modifications (including CoAlation), transient exposure of free protein thiols, and subsequent oxidation of these cysteines by capacitation-associated ROS to drive signalling cascades. This framework is supported by the paradoxical increase in free protein thiols during human sperm capacitation reported by de Lamirande and Gagnon [60]. The conceptual advance of our study is that CoAlation represents a previously unrecognized protective protein thiol modification in spermatozoa. This is particularly relevant as CoASH may emerge as the major protective LMW thiol in human spermatozoa since these cells, unlike somatic cells and bull sperm, have very low GSH levels [2,28].

An interesting observation from our experiments is that protein CoAlation levels responded more prominently to modulation of CoASH biosynthesis under capacitating conditions than under non-capacitating conditions. This differential responsiveness suggests that protein CoAlation becomes dynamically regulated by CoASH biosynthesis once capacitation is triggered. Several features of capacitating spermatozoa may explain this. While PANK activity is subject to feedback inhibition by CoA and acetyl-CoA [61], sperm capacitation significantly increases mitochondrial oxidative phosphorylation [62,63,64], which would rapidly consume acetyl-CoA, reducing the principal allosteric inhibitor of PANK. Furthermore, oxidative conditions promote assembly of the CoA biosynthetic complex [65], explaining why the exogenous pantothenic acid can increase protein CoAlation and impair sperm capacitation (Figure 3b, Figure 5b and Figure 6b). Consistent with this model, pantothenic acid supplementation had no discernible effect on non-capacitated spermatozoa, in which mitochondrial activity is low, and PANK feedback inhibition would remain intact. During sperm capacitation, a process that requires high energy, CoASH is mainly involved in the activation of energy-associated metabolites (e.g., acetyl-CoA) and is not available for protein CoAlation. Then, after removal of CoA from key proteins, the free thiols would be exposed to the oxidation driven by capacitation. This differential responsiveness during capacitation is consistent with the behaviour expected of an intrinsic decapacitation mechanism whose regulatory constraints are lifted only when capacitation-associated signals appear.

Our data show that PANK2 is predominantly abundant in the sperm midpiece (Figure 2c), consistent with its mitochondrial localization in human cells [46]. However, its detection across cytosolic, Triton-soluble, and Triton-insoluble sperm fractions by Western blot, and the extension of immunocytochemistry signal into the proximal principal piece, may have more than one explanation. First, sperm mitochondria are notoriously difficult to isolate cleanly by detergent fractionation. They are helically wrapped around the axoneme and mechanically integrated with outer dense fibers, stabilized by a keratinous mitochondrial capsule and sub-mitochondrial reticulum that physically anchor them to the cytoskeletal scaffold [66]. Triton X-100 extraction may therefore not produce a strict separation between mitochondrial and other compartments, and the partitioning we observe could reflect partial membrane solubilization of a mitochondrially anchored protein. This is consistent with the study by Baker et al. [67], who identified numerous oxidative phosphorylation proteins in the Triton-insoluble sperm fraction even though these proteins originate in mitochondria. Second, the polyclonal antibody we used (HPA008440) may have some cross-reactivity with related PANK family members, particularly the cytosolic isoform PANK3, which could contribute to signal beyond the midpiece. Third, Leonardi et al. [68] demonstrated that the mitochondrial targeting sequence of PANK2 is a primate-specific acquisition: in non-primate mammals, PANK2 is natively cytosolic, possesses identical biochemical regulatory properties to the human enzyme, and is fully functional. This finding establishes that PANK2 does not require mitochondrial localization for enzymatic activity. Our data therefore indicate that PANK2 is present in, but not necessarily confined to, the mitochondria in human spermatozoa.

The properties of protein CoAlation described here raise broader questions about its potential roles across spermatogenesis and early development. The strong CoAlation signal in the post-acrosomal region raises the possibility that CoAlation contributes to stabilizing structures associated with the protamine disulfide network that compacts the sperm nucleus. Defects in CoASH biosynthesis disrupt spermiogenesis in mice, leading to azoospermia [45], and the formation of protamine disulfide cross-linking is a major feature of spermiogenesis [69]. Together, these observations suggest that CoASH-dependent processes, including CoAlation, contribute to proper nuclear compaction during spermiogenesis. A reversible disulfide modification, such as CoAlation, could, in principle, support the highly cross-linked protamine disulfide structure. Conversely, if CoAlation is present in this nuclear structure, the global decline in CoAlation levels (deCoAlation) we observed during capacitation may facilitate the chromatin loosening that precedes fertilization [70]. However, the enzyme(s) responsible for deCoAlation have yet to be identified. By analogy to protein deglutathionylation, which is catalyzed by oxidoreductases known as glutaredoxins (of which five isoforms exist in mammalian cells), deCoAlation is expected to involve dedicated oxidoreductases, yet to be identified, called ‘CoAredoxins’ [29]. The existence of such enzymes in spermatozoa is plausible given that these cells already express specialized thiol-disulphide oxidoreductases. Single-cell transcriptomic and immunohistochemical data from the Human Protein Atlas confirm that TXNRD3, the cognate reductase for the sperm-specific thioredoxins Sptrx-1 and Sptrx-2 [71], is expressed at the protein level in cells of the human seminiferous ducts [72]. Similarly, glutaredoxin 2 (GRX2), which exists as testis-specific cytosolic isoforms (GRX2b and GRX2c) distinct from its ubiquitous mitochondrial form, is the most highly expressed glutaredoxin in human spermatids [73]. Whether these existing thiol-disulphide oxidoreductases can catalyze deCoAlation, or whether a distinct CoAredoxin system exists, remains an important open question and requires further investigation.

From a clinical perspective, our findings are relevant to the ongoing search for functional biomarkers of male fertility. Routine semen analysis does not assess capacitation or the integrity of the signalling events that ensure successful fertilization. These processes may be impaired in men with idiopathic infertility despite having normal semen parameters. Our findings show that the decline in protein CoAlation under capacitating conditions could serve as a readout of the integrity of the redox signalling pathways that govern capacitation. Failure of CoAlation to decrease, or persistence of high CoAlation levels under capacitating conditions, may therefore identify men with defects in capacitation. Conversely, in men whose antioxidant capacity is too compromised to sustain a CoAlation response, baseline CoAlation would be low, removing this intrinsic decapacitation mechanism and producing prematurely capacitated spermatozoa [17]. CoAlation-based measurements could provide a functional assessment of sperm fertilizing potential and complement existing clinical evaluations of male infertility, particularly in men classified as having idiopathic infertility.

These results should, however, be interpreted with several limitations in mind. We assessed only the rate-limiting enzymes PANK2 and COASY and did not examine the full complement of CoASH biosynthesis enzymes. Resolving whether the extra-midpiece PANK2 signal represents a fractionation artifact, antibody cross-reactivity with a related isoform, or an extra-mitochondrial pool would require electron microscopy, isoform-specific antibodies, or proteomic approaches that exceed the goal of the present study. Pharmacological modulators may have off-target effects, although no changes in viability or baseline motility were observed. Finally, all experiments were performed under a single in vitro capacitation model using FCSu, which may not fully recapitulate the complex in vivo environment in the Fallopian tubes. Future studies should leverage other capacitation models, including the induction of capacitation with follicular or oviductal fluid.

## 5. Conclusions

This study provides the first evidence that protein CoAlation may represent a novel intrinsic decapacitation mechanism in human spermatozoa. Our findings show that CoAlation is present at high levels in several protein bands in non-capacitated spermatozoa and is diminished under capacitating conditions. Inhibitors of CoASH biosynthesis lowered CoAlation levels and promoted the redox-sensitive phosphorylation cascades and functional outcomes of capacitation, whereas supplementation of CoASH precursors enhanced CoAlation and impaired these processes. These observations are consistent with a model in which high CoAlation restrains premature capacitation signalling and that its decline is required to permit sperm capacitation. Future work should focus on identifying the specific CoAlated protein targets and evaluating whether CoAlation levels under capacitating conditions can serve as a biomarker of sperm fertilizing potential in men with idiopathic infertility.

## Data Availability

Data are contained within the article.

## Supplementary Materials

The following supporting information can be downloaded from https://www.mdpi.com/article/10.3390/antiox15050600/s1: Figure S1: (a,b) Immunolocalization of protein CoAlation, PANK2, and CoASY in non-permeabilized human spermatozoa, S1c Representative CASA figures for effect of PANK2i and pantothenic acid; Figure S2: Effect of HOPan on sperm motility, viability, and capacitation-associated phosphorylation events; Figure S3: Uncropped immunoblots for subcellular fractionation of protein CoAlation, PANK2, and CoASY, Effect of PANK2i & pantothenic acid on protein CoAlation; Figure S4: Uncropped immunoblots for the effect of PANK2i, pantothenic acid and HOPan on PKA substrate phosphorylation; Figure S5: Uncropped immunoblots for the effect of PANK2i, pantothenic acid and HOPan on tyrosine phosphorylation.

## Abbreviations

The following abbreviations are used in this manuscript:

AKT	Protein Kinase B
AR	Acrosome Reaction
AURKA	Aurora A Kinase
BWW	Biggers–Whitten–Whittingham medium
CASA	Computer-Assisted Sperm Analysis
CoASH	Coenzyme A (free thiol form)
CoASY	Coenzyme A Synthase
CRISP1	Cysteine-Rich Secretory Protein 1
DTT	Dithiothreitol
ECL	Enhanced Chemiluminescence
ERK	Extracellular Signal-Regulated Kinase
FCSu	Fetal Cord Serum Ultrafiltrate
FITC	Fluorescein Isothiocyanate
GRX2	Glutaredoxin 2
GSH	Glutathione
HBS	HEPES-Buffered Saline
HOPan	Hopantenate (pantothenic acid analogue/PPCS inhibitor)
HOS	Hypo-Osmotic Swelling test
H_2_O_2_	Hydrogen Peroxide
IRB	Institutional Review Board
NEM	N-Ethylmaleimide
NO	Nitric Oxide
NYD-SP27	Not Yet Determined Sperm Protein 27
O_2_^−^	Superoxide Anion
PANK2	Pantothenate Kinase 2
PANK2i	Pantothenate Kinase 2 Inhibitor
PBS	Phosphate-Buffered Saline
PI3K	Phosphoinositide 3-Kinase
PKA	Protein Kinase A
PKA-R	Regulatory Subunit of Protein Kinase A
PKC	Protein Kinase C
PPCS	Phosphopantothenoylcysteine Synthetase
PPCDC	Phosphopantothenoylcysteine Decarboxylase
pPKAS	Phospho-PKA Substrate
PSA-FITC	Pisum sativum Agglutinin conjugated with FITC
pTYR	Protein Tyrosine Phosphorylation
RONS	Reactive Oxygen and Nitrogen Species
ROS	Reactive Oxygen Species
SDS-PAGE	Sodium Dodecyl Sulfate-Polyacrylamide Gel Electrophoresis
SEM	Standard Error of the Mean
Sptrx-1/2	Sperm-Specific Thioredoxins 1 and 2
TTBS	Tween-Tris Buffered Saline
TXNRD3	Thioredoxin Reductase 3
WHO	World Health Organization
