# Supplementary material for: Levels of Protein CoAlation Regulate Redox Signaling Events of Human Sperm Capacitation
Source: Antioxidants (Basel). 2026 May 9;15(5):600. doi: 10.3390/antiox15050600 (PMC13203821; doi:10.3390/antiox15050600)
Supplement: Supplementary file 1 [file antioxidants-15-00600-s001.zip › antioxidants-4291720-supplementary.pdf]

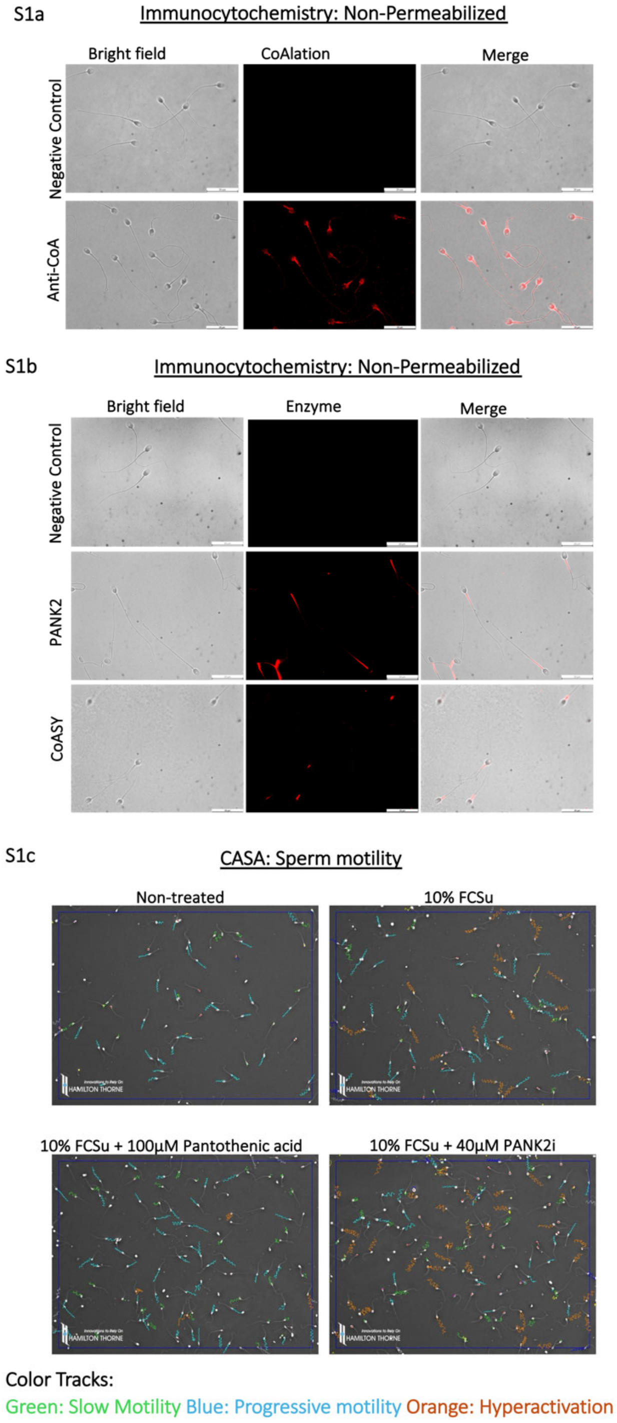

Supplementary Figure S1. (a) Representative immunofluorescence images showing localization of protein CoAlation in non-permeabilized spermatozoa. The staining pattern is consistent with that observed in permeabilized cells (Figure 1b). (b) Representative immunofluorescence images showing localization of PANK2 and CoASY in non-permeabilized spermatozoa. The staining pattern is comparable to that observed in permeabilized cells (Figure 2c). Negative controls processed without primary antibody showed no detectable signal. Images were acquired at 63× magnification; scale bar = 20 µm. (C) Representative CASA figures for the motility effects of PANK2i and pantothenic acid. Blue tracks represent progressive motility, orange tracks indicate hyperactive motility, and green tracks indicate slow motility.

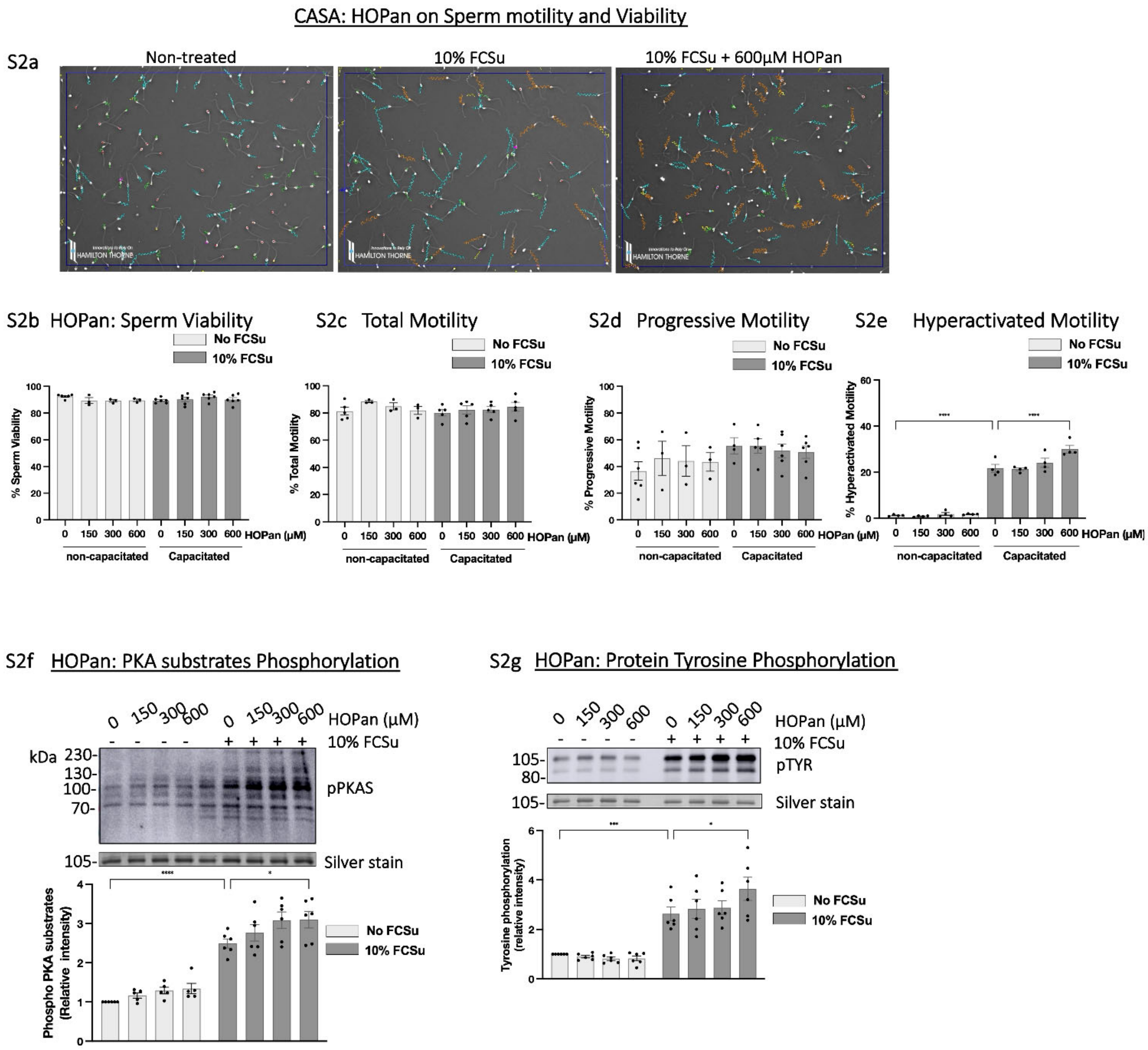

Supplementary Figure S2. (a) Representative CASA images and (b-e) quantitative analysis of sperm motility parameters and viability under non-capacitating and capacitating conditions with increasing concentrations of HOPan. Blue tracks represent progressive motility, orange tracks indicate hyperactive motility, and green tracks indicate slow motility. (f) Western blot and densitometric quantification of pPKAS levels in spermatozoa after 30 min incubation in 10% FCSu ± HOPan (0–600 µM). (g) Western blot and densitometric quantification of pTYR levels after 3.5 h incubation in 10% FCSu ± HOPan (0–600 µM). All densitometric values were normalized to silver stain loading control and then to non-capacitated controls. Data are presented as mean ± SEM; individual data points represent different donors (n = 6). Data were analyzed using ANOVA followed by Bonferroni post hoc test. Significant differences are indicated as *p < 0.05, **p < 0.01, ***p < 0.001, ****p < 0.0001.

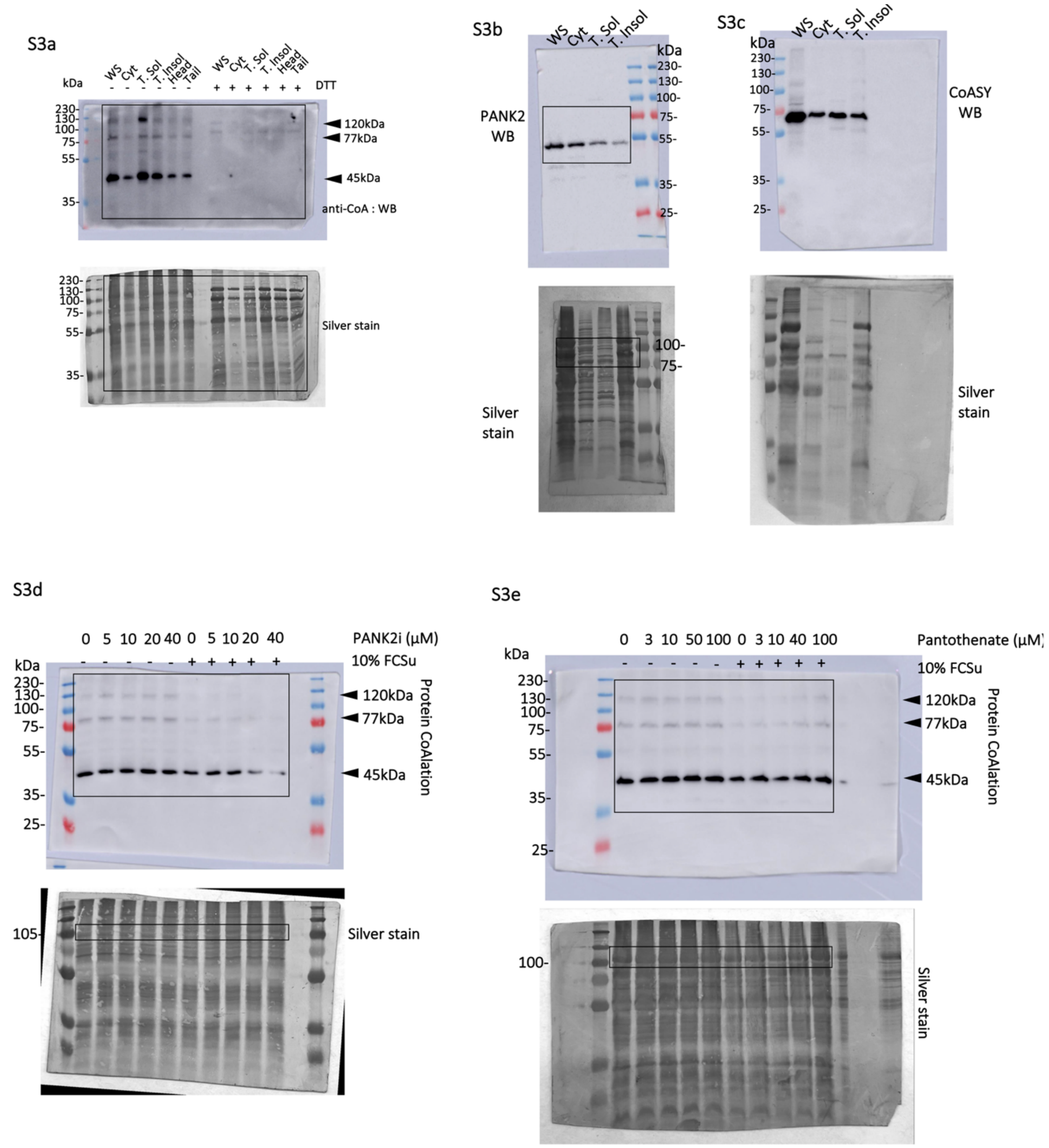

Supplementary Figure S3. Uncropped immunoblots (a) Full-length immunoblot for protein CoAlation across subcellular fractions. (b) Full-length immunoblot for PANK2 across subcellular fractions. (c) Full-length immunoblot for CoASY across subcellular fractions. (d) Full-length immunoblot showing the effect of PANK2i on protein CoAlation levels during capacitation. (e) Full-length immunoblot showing the effect of pantothenic acid on protein CoAlation levels during capacitation.

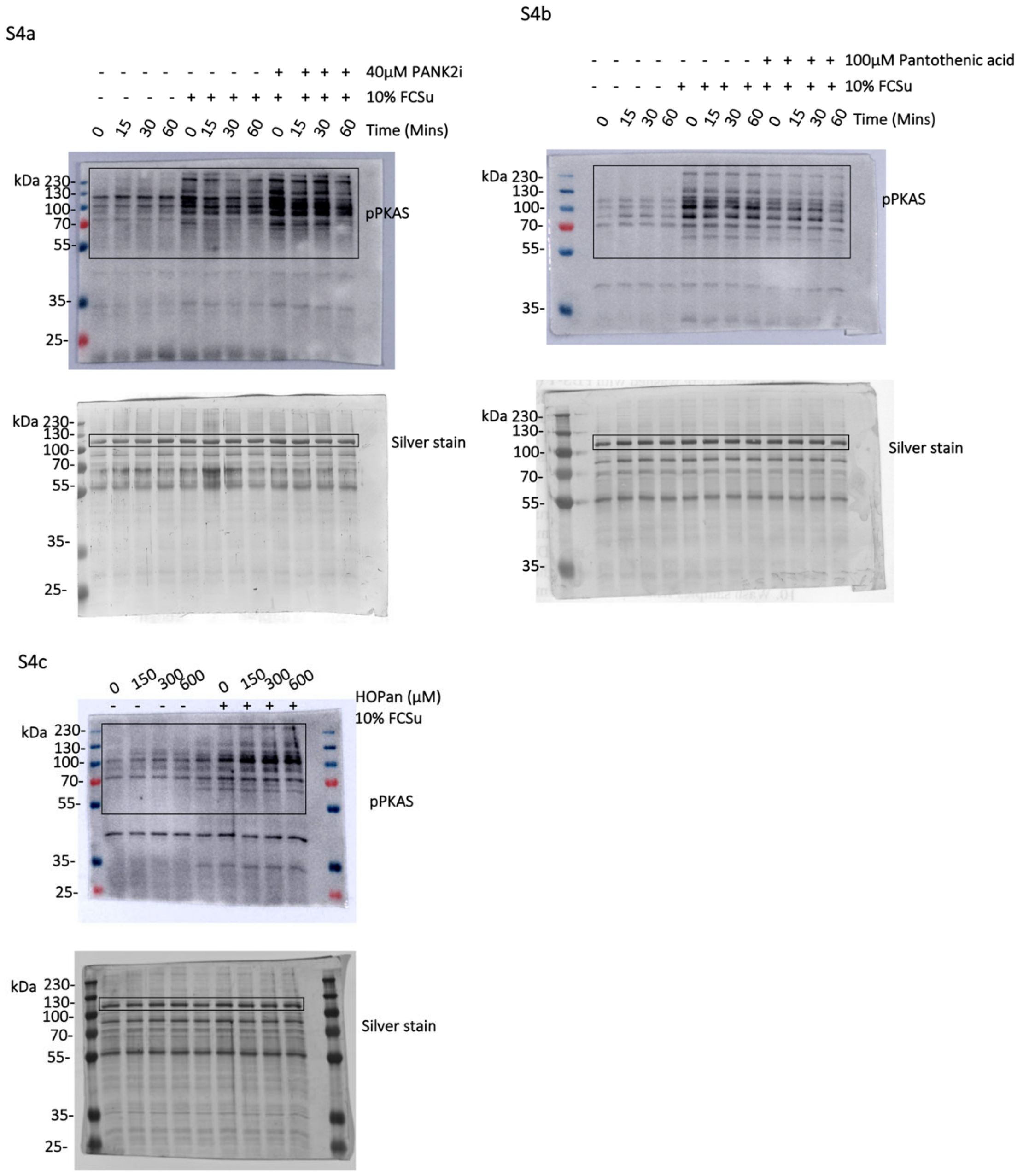

Supplementary Figure S4. Uncropped immunoblots for the effect of PANK2i (a) pantothenic acid (b) and HOPan (c), on PKA substrate phosphorylation (pPKAS) during capacitation.

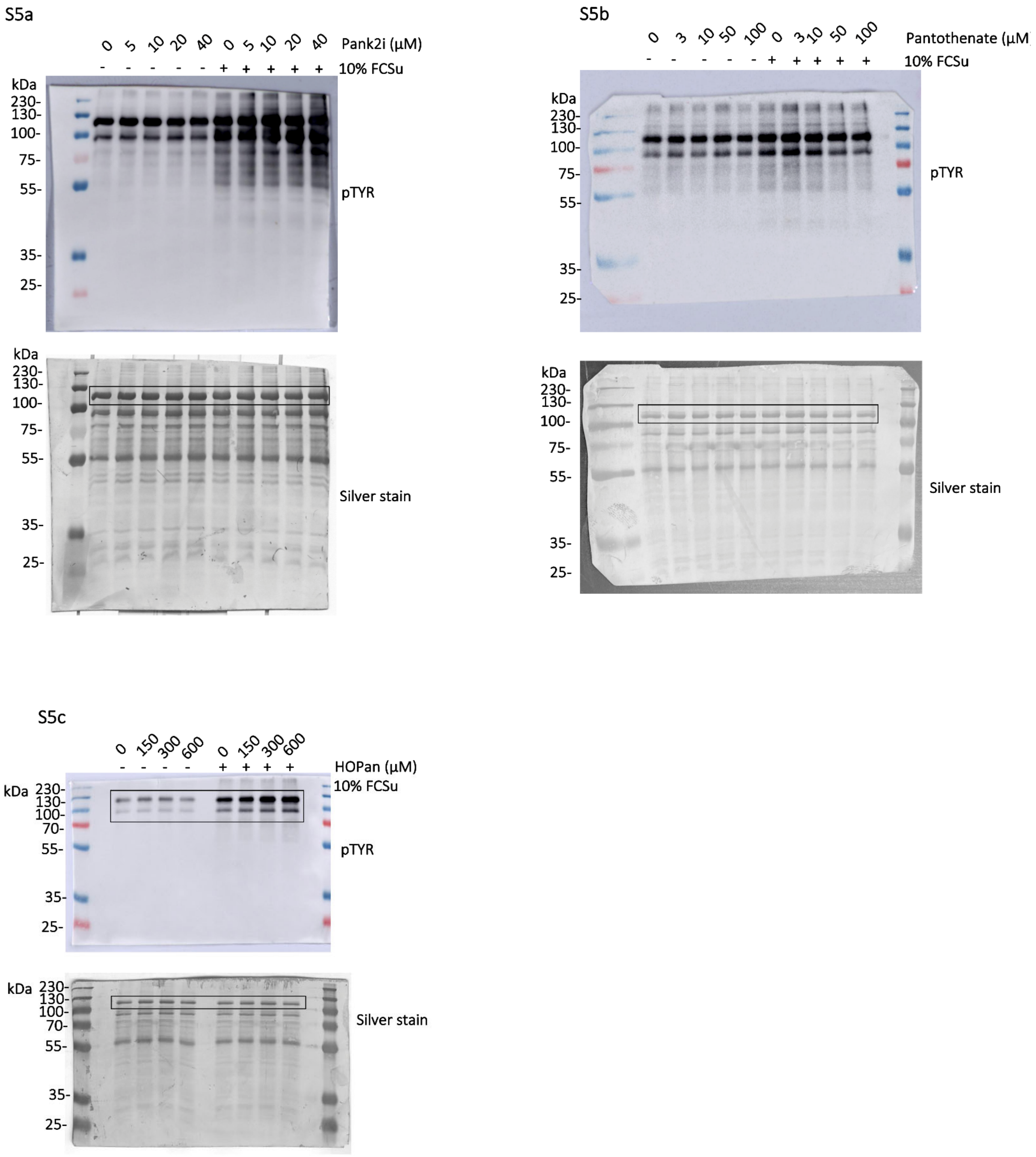

Supplementary Figure S5. Uncropped immunoblots for the effect of PANK2i (a) pantothenic acid (b) and HOPan (c), on tyrosine phosphorylation (pTYR) during capacitation.
